# Photobleaching Effect on the Sensitivity Calibration at 638 nm of a Phosphorus-Doped Single-Mode Optical Fiber Dosimeter

**DOI:** 10.3390/s24175547

**Published:** 2024-08-27

**Authors:** Fiammetta Fricano, Adriana Morana, Martin Roche, Alberto Facchini, Gilles Mélin, Florence Clément, Nicolas Balcon, Julien Mekki, Emmanuel Marin, Youcef Ouerdane, Aziz Boukenter, Thierry Robin, Sylvain Girard

**Affiliations:** 1 Laboratory Hubert Curien, Université Jean Monnet, 42000 Saint-Etienne, France; fiammetta.fricano@univ-st-etienne.fr (F.F.); adriana.morana@univ-st-etienne.fr (A.M.); martin.roche@univ-st-etienne.fr (M.R.); alberto.facchini@univ-st-etienne.fr (A.F.); emmanuel.marin@univ-st-etienne.fr (E.M.); ouerdane@univ-st-etienne.fr (Y.O.); aziz.boukenter@univ-st-etienne.fr (A.B.); 2 CNES, 31400 Toulouse, France; florence.clement@cnes.fr (F.C.); nicolas.balcon@cnes.fr (N.B.); julien.mekki@cnes.fr (J.M.); 3 Exail, 22300 Lannion, France; gilles.melin@exail.com (G.M.); thierry.robin@exail.com (T.R.); 4 Department of Electrical and Information Engineering, Politecnico di Bari, 70125 Bari, Italy; 5 Institut Universitaire de France (IUF), Ministère de l’Enseignement Supérieur et de la Recherche, 1 rue Descartes, 75005 Paris, France

**Keywords:** dosimetry, photobleaching, LUMINA, phosphorus

## Abstract

We investigated the influence of the photobleaching (PB) effect on the dosimetry performances of a phosphosilicate single-mode optical fiber (core diameter of 6.6 µm) operated at 638 nm, within the framework of the LUMINA project. Different irradiation tests were performed under ~40 keV mean energy fluence X-rays at a 530 µ Gy(SiO_2_)/s dose rate to measure in situ the radiation-induced attenuation (RIA) growth and decay kinetics while injecting a 638 nm laser diode source with powers varying from 500 nW to 1 mW. For injected continuous power values under 1 µW, we did not measure any relevant influence of the photobleaching effect on the fiber radiation sensitivity coefficient of ~140 dB km^−1^ Gy^−1^ up to ~30 Gy. Above 1 µW, the fiber radiation sensitivity is significantly reduced due to the PB associated with the signal and can decrease to ~80 dB km^−1^ Gy^−1^ at 1 mW, strongly affecting the capability of this fiber to serve as a dosimeter-sensitive element. Higher power values up to 50 µW can still be used by properly choosing a pulsed regime with periodic injection cycles to reduce the PB efficiency and maintain the dosimetry properties. Basing on the acquired data, a simple model of the photobleaching effect on a coil of the investigated fiber is proposed in order to estimate its sensitivity coefficient evolution as a function of the cumulated dose and its fiber length when injecting a certain laser power. Additional studies need to investigate the influence of the temperature and the dose rate on the PB effects since these parameters were fixed during all the reported acquisitions.

## 1. Introduction

In recent years, advances in space exploration have led to an increasing attention on dosimetry, i.e., the measurement of radiation doses, in space environments. Exposure to cosmic radiation poses significant risks to astronauts, spacecraft, and sensitive equipment during long-duration missions [1,2,3,4,5]. The need for more comprehensive understanding of the radiation field associated with longer-period, onboard-manned missions make radiation monitoring essential. Both active and passive dosimetry have been and continue to be carried out on the International Space Station (ISS) using a variety of detector systems [6,7,8]. Among the promising new technologies for radiation measurement, optical fibers have shown great potential, especially in mixed-field radiation environments [9,10,11]. Fiber optic dosimeters offer several advantages over traditional methods, including their small size, immunity to electromagnetic interference, and real-time monitoring capabilities. In general, they can exhibit a more or less intense sensitivity to radiations. This feature physically comes from macroscopic effects such as radiation-induced effect (RIA), mainly causing a decrease in the transmitted light intensity [9,12]. By characterizing this response under radiation, it is possible to correlate the collected signal from the optical fiber with physical quantities such as dose and dose rate of a space environment. The microscopic origin of such effect is due to the generation under radiation of point defects that absorb part of the transmitted light [13]. The absorption bands associated with these defects contribute to an increase in the intrinsic pre-existing attenuation levels at specific wavelengths [14]. These centers are extremely dependent on the nature of the elements (i.e., dopants) incorporated in the silica matrix during the optical fiber manufacturing process. Among the possible dopants, phosphorus seems to exhibit the best dosimetry properties in terms of RIA, defining the class of radiation-sensitive fibers [9,15,16]. This feature appears very promising to characterize different radiation sources, and spacing from neutrons [17] and gamma-rays [18] to protons and mixed radiation environments [19]. The phosphorus dopant has been widely studied to better characterize the related radiation-induced point defects [20], especially in the near-infrared (NIR) and ultraviolet-visible (UV–vis) domains. The P1 defect was proven to be principally responsible for the NIR absorption, whereas the phosphorus oxygen hole center (POHC) is implied mainly in the VIS domain [21]. The unpaired electron of such a configuration can be on a phosphorus non-bridging oxygen (metastable POHC) or be located between two non-bridging oxygen atoms (stable POHC) [13,22]. The related absorption bands are centered at 2.5 eV and 5.3 eV [20]. Regarding their dosimetry properties, a previous study proved an extreme radiation sensitivity (=0.5 dB m^−1^ Gy^−1^) in the visible range, with a RIA response > 50 dB/m up to 100 Gy for wavelengths shorter than 650 nm [23], being ~125 times higher than the one found for the NIR range: ~4 dB km^−1^ Gy^−1^ [24]. This information reveals the potential exploitation of a very low-dose rate detection, especially in the UV–vis domain, compared to the NIR range. Moreover, no relevant temperature and dose rate dependences have been observed, at least up to 300 °C and in the 1–50 Gy/s range [25]. Their potential as radiation sensors in the VIS range was also suggested elsewhere [26,27] for detection of low-dose gamma radiation levels, by using a multimode (MM) fiber. If, on one hand, MM fibers are easy to handle, it is more difficult, compared to single-mode (SM) fibers, to very precisely measure the radiation-induced attenuation level over long period. Consequently, SM fiber could allow a better stability of the power transmitted during the mission and therefore could detect smaller doses. When performing the RIA measurements, the presence of photobleaching (PB) [10,28,29], basically consisting of a metastable defects recovery induced by the light absorption with the same operational wavelength, has to be considered. This will strongly affect fibers with smaller core sizes since power density is inversely proportional to the sensitive area (i.e., doped core region). The combination of such studies paves the way for the development of a two-channel dosimeter optical fiber-based method to monitor the ISS radiation levels [30,31]. Using in parallel two different SM P-doped optical fibers, one operating in the visible domain and the other in the infrared one, it is possible to compare the results and combine them to detect the lowest doses. The so-called LUMINA dosimeter has been operating since August 2021, and some data from ISS regarding solar particle storms have been already collected and analyzed [32]. This paper reports a preliminary study that was conducted before the official launch and that contributed to the calibration and design of the LUMINA visible channel. In particular, we studied the potential of the SM fiber at 638 nm since it was found that the spectral range around 600 nm represents an interesting wavelength domain for its dosimetric properties, as reported in [33]. At ~600 nm, we found a good compromise between the dose dependence linearity (up to 100 Gy) and a high sensitivity coefficient. In addition, we quantified a reduced temperature and dose rate dependence compared to the others wavelength in the VIS domain. This motivated the study’s focus on such a spectral range, and the availability of (relatively powerful) optical sources led to the choice of a laser diode at 638 nm as the operational wavelength. In order to investigate the influence of the photobleaching effect, we injected a laser diode light between 500 nW and 1 mW power values and observed the associated changes in the RIA growth and decay kinetics. In this way, it is possible to have an idea of which power range can be used when performing RIA measurements for dose monitoring to ensure that the PB is minimized as well as the associated uncertainty of the retrieved dose. On the other hand, the results from a systematic study were used to create a dedicate model of the sensitivity evolution of the fiber coil used as the sensitive element of the dosimeter as a function of the cumulated dose when a certain light power within the studied range is injected. This model takes into account the photobleaching at 638 nm in order to calculate the losses along a given fiber length of the selected single-mode phosphosilicate optical fiber and thus the related RIA coefficients.

## 2. Materials and Methods

- LUMINA fiber sample

The tested sample is the latest version of the optical fiber developed by EXAIL (Lannion, France) [34], specially designed for the LUMINA dosimeter. It is SM at the operational wavelength of 638 nm, and it presents an external cladding with an outer diameter of 80 µm (to reduce the volume of the sensitive fiber coil within LUMINA) and a phosphorus-doped core with a 6.6 µm diameter. With the acrylate coating, the outer fiber diameter is 128 µm. The intrinsic losses (before irradiation) at 600 nm are less than 12 dB/km and they were considered in the data analysis. In the following table, we report the useful specifics given by the manufacturer.

**Figure 1:**
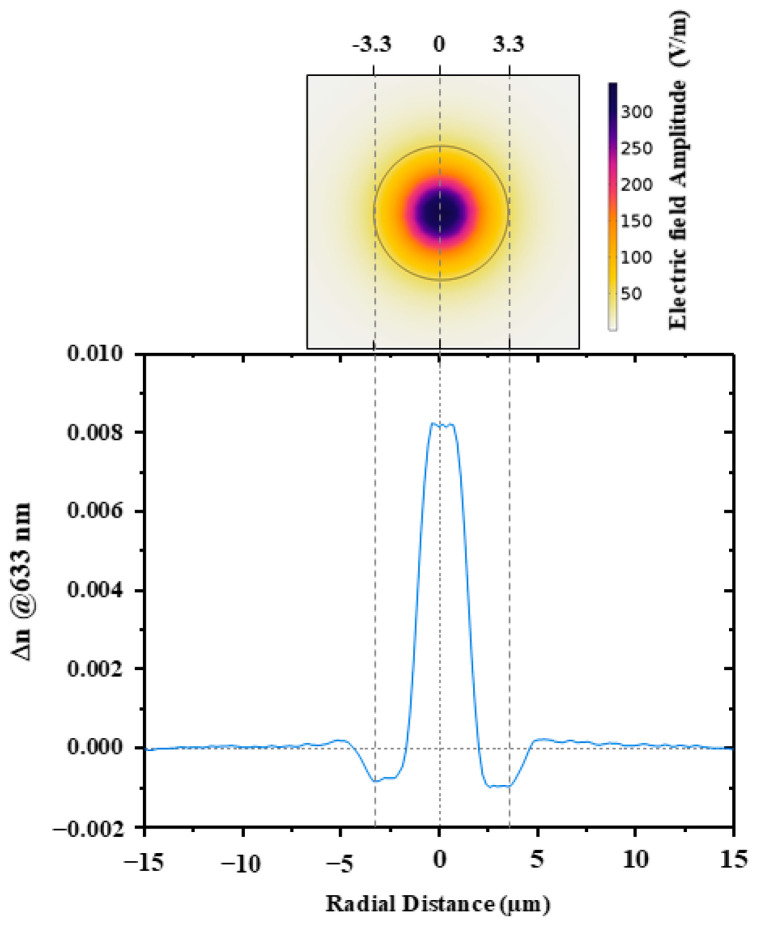
Refractive index profile measured at 633 nm for the LUMINA P-doped optical fiber. On the top, the simulated 2D electric field distribution for the fundamental mode at λ = 638 nm. Simulations were carried out using COMSOL Multiphysics software, version is 5.2.1.152 [35].

To give a complete panorama of the fiber characteristics, we report in Figure 1 the refractive index profile of this fiber measured at 633 nm, showing a maximum refractive index difference Δn between the core and cladding of ~0.008. In addition, the amplitude of the electric field is analyzed in the “Mode Analyses” study from COMSOL Multiphysics [35], from the fiber structure and parameters shown in Table 1. These results of the study confirmed the single-mode behavior propagation in this optical fiber at 633 nm.

- Experimental Setup

**Figure 2:**
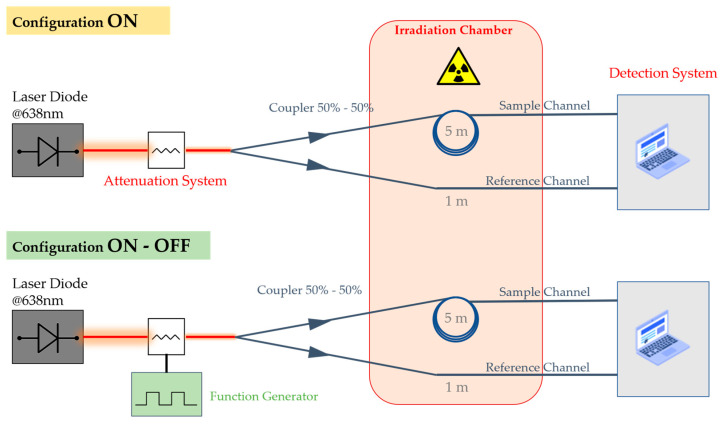
Scheme of the set-up used for the two parallel acquisition methods: (**up**) configuration ON; (**down**) configuration ON–OFF.

All the tests were performed at the Hubert Curien Laboratory (Saint-Etienne, France), using the LabHX X-ray irradiation machine. Its tungsten target is powered by a 100 kV voltage able to produce photons with a ~40 keV mean energy fluence [36]. With this generated spectrum, and by placing the samples as far as possible from the X-ray source, the lowest achievable dose rate without additional shielding is ~530 µGy(SiO_2_)/s. Figure 2 reports a scheme of the setups used for two different configurations, which are later discussed in the text. For each configuration, we planned two different channels (double-beam technique). The first one was constituted by a 9 m long fiber coil of the tested P-doped SM fiber, whose ~4 m were needed to transport the signal inside and outside the irradiation chamber, and 5 m were coiled and uniformly irradiated. The second channel, instead, acted as reference channel, constituted by ~5 m length of the same optical fiber, of which only 1 m was irradiated in order to monitor and compensate any laser diode related fluctuations and the losses induced on the ~4 m length used as transport fiber. Indeed, no dedicated transport fiber was used in order to avoid attenuations related to splice presence. So, the fiber itself was employed to connect the fiber under test to the instrumentation, but these parts of the optical fibers were carefully shielded with copper tubes, limiting the irradiation induced losses. To ensure an equal signal repartition, a 50/50 coupler was inserted in the setup that was able to equally split the injected power at 638 nm.

To investigate the photobleaching effect, we repeated various irradiation tests by injecting, into the fibers under test, different 638 nm light signal powers for each run. As explained in the introduction, the selected range of powers (<1 mW) was motivated by previous preliminary measurements in the visible spectrum, some of them performed in the context of the LUMINA project. In addition, the availability of optical sources enforced the choice to operate at 638 nm, which appeared as the best compromise. The used diode allowed to inject a maximum of ~1 mW into the investigated SM fiber. In fact, different power levels between 500 nW and 1 mW were achieved by either adjusting the power output of the optical source or by using an optical attenuator. This allowed operations at very low power while staying within the laser diode’s operational limits. To study the photobleaching influence, we performed the measurements by using two different configuration types:

- Configuration ON means that the laser is always switched on during all the acquisition time, including the “recovery” period following the end of irradiation. If any photobleaching occurs, this configuration will maximize the effect;
- Configuration ON–OFF involves the use of a function generator able to generate laser pulses for which the laser is ON for 20 s and OFF for 580 s, resulting in periodic sequences in which the laser is injected ~3% of the cycle duration time.

The setup was optimized in order to improve the measurement repeatability and reduce as much as possible any parasitic losses (via splices, curvature loss, etc.). Temperature was controlled during the acquisition runs by placing thermocouples near the samples. In general, all the tests were carried out at room temperature (23 °C ± 3 °C). The detection assembly was constituted by two InGaAs detectors in the VIS range, one for each channel, that are able to receive up to 20 mW and are then processed by a dual-channel optical power meter directly connected to the computer. In this way, it was possible to monitor in real time the signal power in the tested samples and record the radiation-related attenuation of the fibers under test.

## 3. Analysis and Results

Thanks to the employment of two different parallel channels, the reference and the sample one, it was possible to monitor and correct the signal from any laser source fluctuations. The total RIA was obtained by the ratio between the two signals for each injected power. Equation (1) is used to calculate the radiation induced losses for each channel (*Ref* stands for reference and *Sam* for sample):

(1) $${Loss}_{i}\;\left(dB\right)=-10\log\left(\frac{I_{i}\left(t\right)-noise}{I_{i}\left(0\right)-noise}\right),\;\;i=Reference,\;Sample$$

where *I* is the intensity of the transmitted signal measured in mW by the detector at any time *t* of the acquisition, with *t* = 0 as the irradiation start. The noise corresponds to the situation in which the laser source is switched off. The RIA of the fiber under test is then obtained by subtracting the two results for each measured *t* and dividing it for the difference between the length of the two coils, i.e., the reference and the sample ∆L ~ 4 m)), according to Equation (2):

(2) $$RIA\;\left(\frac{dB}{m}\right)=\frac{1}{∆L}\;({Loss}_{Sam}-{Loss}_{Ref})$$

**Figure 3:**
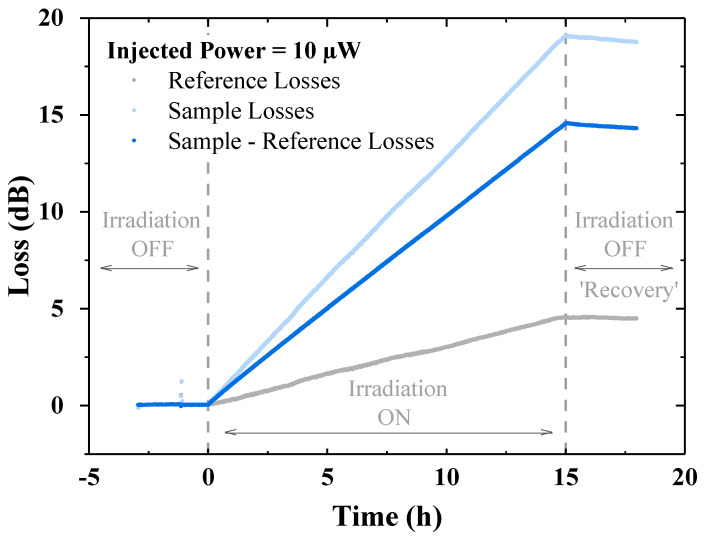
Acquired signal with a 10 µW injected power in the reference (1 m long sample) and sample (5 m long sample) channels. To eliminate the contribution from the laser source fluctuations and the transport fibers losses, the two signals were subtracted. In the figure are also marked the three different acquisition periods. When the irradiation is off, the laser remains switched on.

Figure 3 shows an example of the induced losses for an injected power of 10 µW. The same procedure was performed for each laser power from 500 nW to 1 mW and for both mentioned configurations, ON and ON–OFF, using for each irradiation run a new, not-irradiated sample of the SM fiber. The different measurement steps are also marked to distinguish the moments when there is no irradiation (but the laser is still switched on) and when the X-rays irradiation starts, followed by a few “recovery” hours corresponding to “laser ON, irradiation OFF”.

### 3.1. Configuration ON

**Figure 4:**
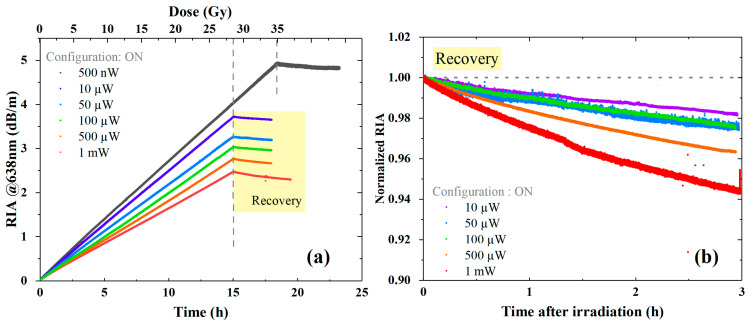
RIA kinetics at 638 nm, in configuration ON, for different injected powers on the tested LUMINA SM fiber, with each curve corresponding to a different irradiation run. (**a**) RIA growth vs. time and dose during ~15 h irradiation, up to a cumulated dose of 28–35 Gy, using a 530 µGy/s dose rate. Dashed lines indicate the irradiation end and the recovery start while the laser source is still switched ON. (**b**) RIA levels normalized by the achieved value at the irradiation end. Decay kinetics of the RIA post irradiation for 3 h; the decay remains limited to 6% in the worst-case scenario (1 mW of injected power light).

Figure 4a reports the RIA obtained at 638 nm up to a dose of 27–35 Gy at different injected powers from 500 nW to 1 mW in the configuration ON, where the laser is injected during all the measurements. We observed a linear dependence of the attenuation levels as a function of the cumulated dose for the particular investigated dose rate. This facilitated the analysis by using a simple linear fit to obtain the related sensitivity coefficients, expressed in dB km^−1^ Gy^−1^. The measurement acquired using an injected power of 500 nW lasted a few hours longer than the others, as we wanted to check that the linearity was proven even for doses higher than the ones initially planned. The graph clearly shows a photobleaching effect since the RIA level decreases as the injected power increases. In any case, the response vs. the dose remains linear, allowing an easy estimation of the sensitivity coefficient. In the same way, the post-irradiation recovery (Figure 4b) is affected by the injected power value, revealing a significative change in the recovery percentage above 500 µW. The recovery when using 500 nW as injected power is not shown since it refers to a recovery starting point from a 35 Gy cumulated dose, whereas the other signals are comparable due to the same 27 Gy received during the ionizing radiation dose. However, 3 h after the end of irradiation, we recovered only 6% of the signal when using 1 mW as the injected power.

### 3.2. Configuration ON–OFF

**Figure 5:**
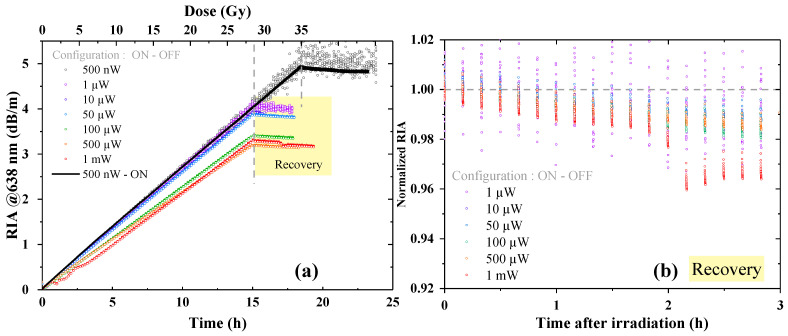
RIA kinetics at 638 nm, in configuration ON–OFF, for different injected powers on the tested LUMINA SM fiber, with each curve corresponding to a different irradiation run. (**a**) RIA growth vs. time and dose during 15 h irradiation, up to a cumulated dose of 27–35 Gy, using a 530 µGy/s dose rate. Dashed lines indicate the irradiation end and the recovery start while the laser source is still in configuration ON–OFF. (**b**) RIA levels normalized by the achieved value at the irradiation end.

Under the same irradiation conditions, we repeated the measurements of the previous section but in configuration ON–OFF. The RIA curves obtained during and after irradiation are shown in Figure 5a, whereas Figure 5b illustrates the RIA normalized by the last point before the end of irradiation and during the recovery phase. The trends are similar to the ones obtained in configuration ON, revealing a positive impact of the increased injected power value on the RIA levels that decreased up to 50 µW. We did not find any strong presence of photobleaching since the RIA levels varied within a 3%. Additionally, the fact that the losses when injecting 500 nW in a continuous (also plotted in Figure 5a) or pulsed regime were superimposed confirms this hypothesis. On the contrary, above 50 µW, we clearly observed a decrease in the RIA levels, meaning that such powers are high enough to induce photobleaching. As a consequence, the recovery post irradiation was also only slightly affected, showing a maximum of 2% recovery after 3 h compared to the 6% obtained in configuration ON. Note that in this case as well we did not compare the recovery signal related to 500 nW power due to the different received doses.

## 4. Discussion

**Figure 6:**
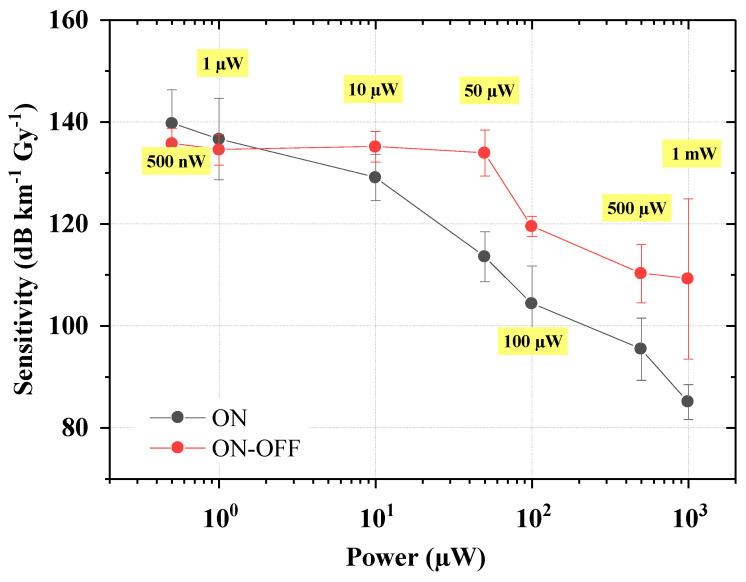
Sensitivity coefficient evolution as a function of the injected laser power, for both configurations ON (black curve) and ON–OFF (red curve). The error bars come from repeatability tests.

The obtained results revealed the presence of the photobleaching effect on the RIA of this phosphosilicate SM optical fiber in the visible domain. RIA at 638 nm is indeed strongly dependent on the injected power, and the provided power levels are able to favor the point defects recombination. As mentioned in the introduction, the absorbing defects in this spectral range are mainly the two versions (stable and metastable) of the POHC. The amplitude of the PB effect can be controlled either by reducing the laser diode power or by reducing the injection time (percentage of time the laser is ON during the tests). It should be noted that in this particular study, we did not investigate any temperature or dose rate dependence or the possible influence of the time the laser remains ON since these parameters were fixed during all the acquisitions. More precisely, for space applications, low dose rates over fairly long periods must be targeted, with cumulative doses of the order of a few tens of Gys. As a consequence, we operated at the minimum dose rate achievable by our facility, but it should be considered that in most space satellites, the dose rate range is even lower [8]. In that case, the photobleaching effect should be even more impactful on the defect’s recombination due to the larger photon fluence seen by the defects. However, in the tested conditions, it was possible to obtain the sensitivity coefficients for each different injected power value by performing a linear fit (forcing the passage to zero) of the RIA value as a function of cumulated dose. The obtained slopes correspond to the sensitivities, expressed in dB m^−1^ Gy^−1^. They are presented in Figure 6 for both configurations, and the numeric values are reported as well in Table 2, together with the associated R^2^, assessing the quality of the performed linear fit.

We noted in both configurations the presence of the photobleaching effect, especially above 50 µW. When injecting a power between 500 nW and 10 µW, only a slight effect evidence was found in the configuration ON–OFF, with a sensitivity variation within 2% from their average. When the photobleaching starts to affect the RIA levels, causing a sensitivity decrease, even the injection time should affect the RIA values and become more and more important as the injected power increases. Indeed, the ratio between the sensitivity coefficients obtained in the two configurations was ~1.16 when using 50 µW, and it gradually reached ~1.28 when 1 mW was injected. Although this discrepancy is not substantial, it suggests that the injection time parameter has to be considered when exploiting the photobleaching positive effect.

- Model of the photobleaching effects in a fiber coil

The obtained results can be exploited to develop a model including photobleaching that is able to calculate the losses inside a given P-doped optical fiber coil when a laser power within the investigated range is injected. The parameters that we can alter are the intrinsic fiber losses, the sample length, and the inject power value (at 638 nm). First, we determined a function able to reproduce the results shown in Figure 6. However, it should be noted that our model will present some limitations since it is restricted to the used experimental conditions, i.e., for a fixed dose rate and temperature, and in addition, it is based on photobleaching powers within the range of 500 nW–1 mW. As a consequence, we assume that there is no noticeable photobleaching effect for injected laser powers below 500 nW since we did not observe any difference between the ON and ON–OFF configurations, and the first appreciable change was noted after the 10 µW injection. Nevertheless, the response under lower dose rates could be more impacted by the power level and the photobleaching thus be observed. On the other hand, we assumed a saturation trend after 1 mW, but some additional measurements are needed to adapt the chosen function to higher powers. In the end, the law describing the sensitivity comes from a fit with a function type such as the following:

(3) $$Sensitivity=A_{1}-\frac{\left(A_{1}-A_{2}\right)}{1+e^{-p(\log\left(x+x_{0}\right)}}$$

**Figure 7:**
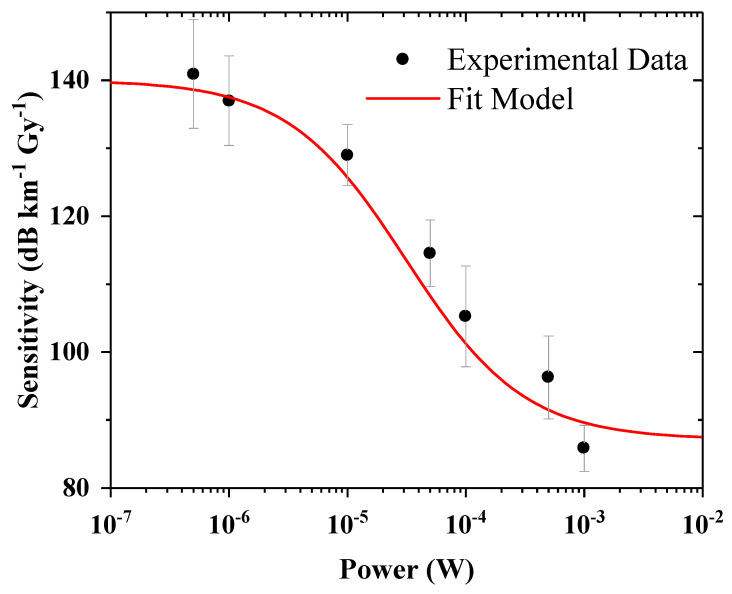
Sensitivity function used to estimate the losses knowing the propagating signal power at 638 nm, starting from the experimental data in configuration ON.

A_1_ and A_2_ correspond to the top and the bottom asymptotic values matching a physical situation in which we have no or no more photobleaching. The fitting parameter *p* is related to the slope of the sigmoidal function. The curve is a function of the injected power *x*, and *x*_0_ is the central point of the flex, equal to 4.5 × 10^−5^ W. The so-obtained calibration curve is shown in Figure 7.

When considering a P-doped fiber coil, the parameters that can be changed are the optical fiber length, which is particularly important when considering the initial attenuation losses and the RIA calculus, and the initial injected power (as long as it is within the range of 500 nW–1 mW).

To calibrate the losses of such a coil as a function of the dose, by considering the photobleaching, we developed an iterative code. The whole coil length was divided into *n* small fiber parts of a length *l*, and for each step of the dose, we calculated the input power injected into each part. By using our photobleaching function, it is possible to calculate the related sensitivity for each *n*-th piece that has already received some radiation, thus impacting its attenuation. Once obtained, the losses were calculated so that the output signal power from the short *n*-th fiber part became the input light of the just-following *n*+1-th piece. It is possible to vary the length of the short-portion fiber for each time interval (and thus the corresponding dose) to obtain a calculus that becomes much more precise as the piece is shortened. The obtained curve will yield the dependence of the dosimeter losses as a function of the deposited dose for the given conditions. The derivative of such result will physically correspond to the sensitivity coefficient, calculated up to a given dose. The curve thus represents the dosimeter calibration curve as evolution of the received dose when subjected to a certain input power.

- Application example

**Figure 8:**
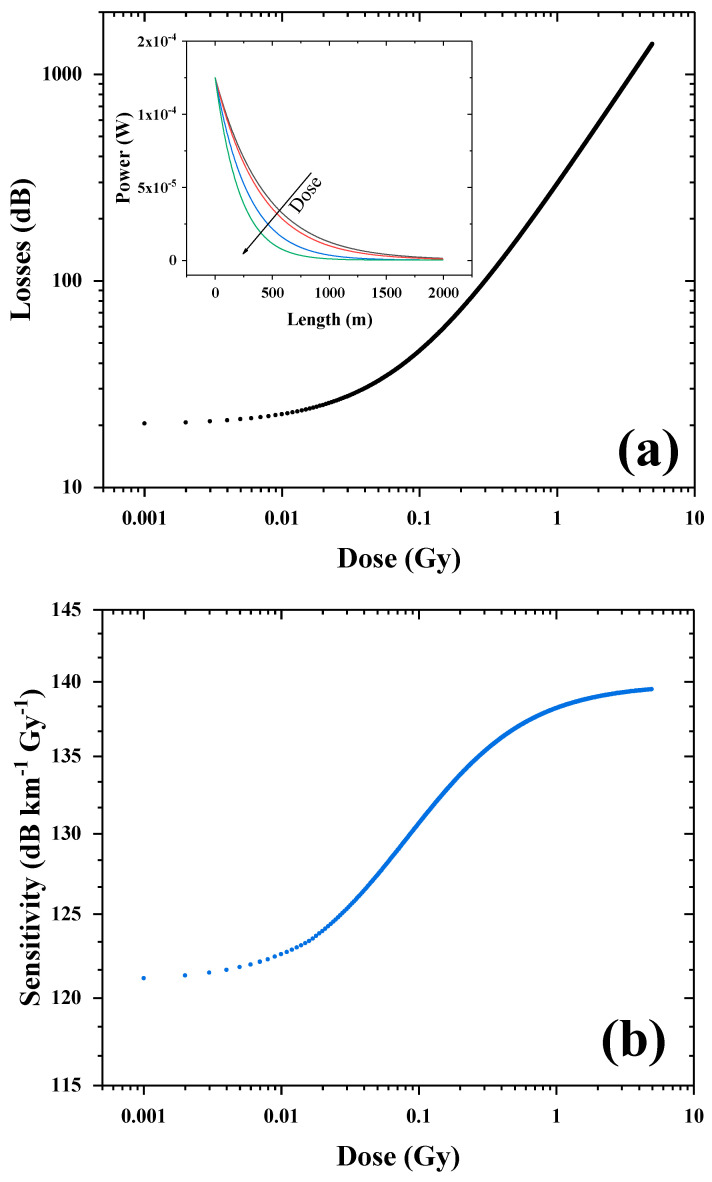
(**a**) Simulated 638 nm losses evolution along a 2 km long optical fiber, up to a 5 Gy cumulated dose, considering the photobleaching effect. In the inset is shown the transmitted power along the fiber length for different intermediary doses between 0–10 Gy, when 125 µW is injected in input. (**b**) Calculated sensitivity coefficient of all the coil as a function of the deposited dose.

As a possible example, we simulated the sensitivity coefficient evolution up to a cumulated dose of 5 Gy for a 2 km long coil of LUMINA P-doped SM optical fiber, where a 125 µW laser power was injected. These choices originated from a particular study in [30], where the IR and VIS calibration of LUMINA fiber was reported for very low dose rate (145 mGy/h = 0.04 mGy/s). The initial 0.25 mW injected power was split into the reference and sample channel (double-beam technique), so we assumed that half (125 µW) of it was actually injected in the 2 km fiber sample. This value is within the application limits of our model, so it was possible to consider the photobleaching effect. The injected power naturally decreased along the fiber length, as shown in the inset of Figure 8, due to intrinsic attenuation $\alpha_{0}$ of ~20 dB/2 km, thus having more impact on the transmission for such long fiber lengths. This effect is more accentuated as the cumulated dose increases, causing a decrease in the transmitted power at a point closer and closer to the beginning of the fiber. The calculated losses (in dB) due to the whole fiber length are shown in Figure 8a, as a function of the dose (in semi-log scale). They remained around the initial attenuation value at least up to 0.1 Gy, then rapidly increased up to 10 Gy. This comes from the impact of the photobleaching effect on the measurement and from its temporal dependence as the cumulative dose increased. Indeed, the PB phenomenon had a stronger impact at the beginning of irradiation, when most of the fiber sample was traversed by sufficiently high optical power to induce a significant decrease (or contribute to maintaining a constant value) in the sensitivity coefficient. The derivative of the losses is reported in Figure 8b, revealing that, in fact, the sensitivity coefficient was ~126 dB km^−1^ Gy^−1^ at least up to 0.01 Gy. As the RIA contribution accumulated together with intrinsic losses, the power propagating in each fiber section decreased significantly, leading to an increase in local sensitivity coefficients, reaching a ~140 dB km^−1^ Gy^−1^ saturation value. This maximum obtained sensitivity value reveals that the photobleaching was not present anymore, according to the calibration curve in Figure 7. Moreover, after 0.05 Gy, we obtained a sensitivity of 131 dB km^−1^ Gy^−1^, which is close to the one found in [30], where the sensitivity was estimated to be (137 ± 6) dB km^−1^ Gy^−1^ under γ-ray exposure up to a ~0.05 Gy cumulated dose. Furthermore, the simulated data were obtained from our results with a 530 µGy/s dose rate, which is more than 10 times higher than the one tested by Di Francesca et al. [30]. Moreover, dose rate dependence could be present for very low dose rates, where the photobleaching can have a stronger influence. Additional data are necessary to implement the code and improve the model behind the sensitivity coefficient evolution.

## 5. Conclusions

In this work, we presented our study on the photobleaching effect on the LUMINA dosimeter based on the use of phosphorus-doped optical fiber operating in single mode at 638 nm. The impact of such an effect was investigated for the RIA levels recorded at 638 nm at the fixed dose rate of 530 µGy(SiO_2_)/s and room temperature. At this wavelength, we achieved a good radiation sensitivity and a linear dependence of RIA versus the dose (at least up to ~30 Gy). Fixing these parameters, evidence of the photobleaching phenomenon was proven by injecting different signal powers into the fiber. In particular, powers lower than 10 µW were not enough to induce a recombination of the P-related defects, whereas higher powers effectively favored it. The consequence is a radiation sensitivity decrease due to a reduction in the RIA levels. Particularly crucial also is the injection time, proven by performing cycles of 20 s of laser injection every 600 s. Above 50 µW, the ratio between the sensitivity under a continuous laser injection and the one subjected to a pulsed regime increased as well the laser power increased. This has to be considered when planning the dosimeter calibration. Additional tests need to be addressed to fully complete such an investigation, for example, varying the temperature or dose rate dependence. Especially for space applications, lower dose rates should be tested, but in our case, this could not be carried out due to the facility limitations. Alternative methods based on the use of some shielding layers are under study in order to perform experiments for very low dose rate detections. In any case, these results can be useful to build a dedicate model able to predict the sensitivity coefficient of a P-doped optical fiber coil when a certain power is injected into it. In addition, the proposed methodology can be employed for other types of optical fibers or other wavelengths. A similar study is in progress for a larger-core multi-mode phosphosilicate fiber that should be more robust against photobleaching in terms of dosimetry properties. Indeed, in that case, due to the larger core size, higher powers are necessary to reach the same power density in the core and then induce a similar photobleaching effect.

## Figures and Tables

**Table 1:** Characteristics of the LUMINA Phosphorus-Doped Single-Mode fiber, given by EXAIL [34].

Fiber Parameter	Value
Numerical Aperture	0.17 ± 0.1
Cut-off Wavelength	~<600 nm
Core Diameter	6.6 µm
Cladding Diameter	80 ± 1 µm
Coating Diameter	128 ± 3 µm
Losses @600 nm	<12 dB/km

**Table 2:** Sensitivity coefficients variation and related R^2^ for each injected power and for both ON and ON–OFF configurations.

Injected Power (µW)	Sensitivity (dB km^−1^ Gy^−1^) ON	R^2^	Sensitivity (dB km^−1^ Gy^−1^) ON–OFF	R^2^
0.5	141	0.993	136	0.993
1	137	0.997	134	0.995
10	129	0.998	135	0.991
50	114	0.999	133	0.988
100	104	0.999	119	0.979
500	96	0.998	110	0.992
1000	85	0.999	109	0.995

## Data Availability

The raw data supporting the conclusions of this article will be made available by the authors on request.
